# A Bearing Fault Diagnosis Method Based on a Residual Network and a Gated Recurrent Unit under Time-Varying Working Conditions

**DOI:** 10.3390/s23156730

**Published:** 2023-07-27

**Authors:** Zheng Wang, Xiaoyang Xu, Yu Zhang, Zhongyao Wang, Yuting Li, Zhidong Liu, Yuxi Zhang

**Affiliations:** 1 School of Mechanical and Electrical Engineering, Shandong Jianzhu University, Jinan 250101, China; 202007101154@stu.sdjzu.edu.cn (X.X.); 202007101149@stu.sdjzu.edu.cn (Y.Z.); 202007101151@stu.sdjzu.edu.cn (Z.W.); 202007101156@stu.sdjzu.edu.cn (Y.L.); 202007101285@stu.sdjzu.edu.cn (Z.L.); 2 School of Management Engineering, Shandong Jianzhu University, Jinan 250101, China; 202102102137@stu.sdjzu.edu.cn

**Keywords:** data-driven, fault diagnosis, GRU, ResNet, time-varying working condition

## Abstract

The diagnosis of bearing faults is an important guarantee for the healthy operation of mechanical equipment. Due to the time-varying working conditions of mechanical equipment, it is necessary to achieve bearing fault diagnosis under time-varying working conditions. However, the superposition of the two-dimensional working conditions of speed and acceleration brings great difficulties to diagnosis via data-driven models. The long short-term memory (LSTM) model based on the infinitesimal method is an effective method to solve this problem, but its performance still has certain limitations. On this basis, this article proposes a model for fault diagnosis under time-varying operating conditions that combines a residual network model (ResNet) and a gate recurrent unit (model) (GRU). Firstly, the samples were segmented, and feature extraction was performed using ResNet. We then used GRU to process the information. Finally, the classification results were output through the output network. This model could ignore the influence of acceleration and achieve high fault diagnosis accuracy under time-varying working conditions. In addition, we used t-SNE to reduce the dimensionality of the features and analyzed the role of each layer in the model. Experiments showed that this method had a better performance compared with existing bearing fault diagnosis methods.

## 1. Introduction

As an important component in the process of mechanical operation, bearings play a crucial role [1,2]. Their function is to support mechanical rotating components to reduce friction and wear. Bearing failures can worsen the operating environment of machines, so accurate, efficient, and simple detection of bearing failures [3] is of great significance for improving the equipment’s safety and avoiding unnecessary production losses [4].

Bearings are affected by different loads and environments during use, such as high temperatures during high-speed rotation and poor lubrication during low-speed rotation. In addition, external impacts, vibrations, corrosion, and friction from impurities can cause changes in the bearings’ speed and acceleration. Therefore, the detection of bearing faults is often carried out under time-varying operating conditions. A general intelligent fault diagnosis method consists of three steps: data collection, feature extraction, and fault recognition. The data collection part is the foundation of detection, plays a fundamental role in determining the accuracy of the subsequent steps, and is the most crucial.

Traditional fault diagnoses usually use signal processing based on frequency and time-domain features and then use various classifiers for signal diagnosis. Typical classifiers include Bayesian classifiers [5,6], support vector machines [7], Random Forest [8] etc. Although these methods are easy to implement, they require specialized knowledge to extract the features and select the classifiers, and their accuracy and reliability also require high data quality and signal characteristics. For example, Zhang et al. [7] proposed an intelligent fault diagnosis method for roller bearings based on a multivariable ensemble of incremental support vector machines. This method can be used to identify different types of faults and their severity. Compared with most algorithms, this algorithm performs better. However, this intelligent fault diagnosis method requires a high sampling frequency during data recording and a data recorder equipped with a low-pass filter in the input stage, thus having relatively high data acquisition requirements. At the same time, this method has a high level of complexity and requires a longer computational time. Xu et al. [9] proposed an early bearing fault detection method called the Ensemble Average of Autocorrelated Envelopes (EAAE). The Ensemble Average of Autocorrelated Envelopes was proposed to identify the occurrence of early faults in bearings, in which the vibration signals are contaminated by the various background noise and noise of phase modulation. The developed methods could suppress noise and tolerate the cyclostationarity of the vibration to achieve the extraction of local weak fault signatures. However, due to certain requirements for signal preprocessing in the early stage, stricter signal processing and preparation are required in practical use. Samanta et al. [10] proposed a bearing fault detection method that combined particle swarm optimization (PSO) with other computational intelligence (CI) technologies. The implementation is relatively simple, and parallel processing based on the PSO method can speed up the calculation. Widodo et al. [11] calculated the statistical features from the measured signals, and used RVM and SVM to diagnose bearing faults. This method has a simple model structure and is easy to implement and adjust. However, the methods above have difficulty collecting samples for practical problems of time-varying speed and acceleration.

In recent years, the use of deep learning models for bearing fault detection has become a popular research direction [12]. Deep learning algorithms have grown increasingly attractive in such rapid applications because of their increased reliabilities and simplicity compared with traditional methods [13]. Furthermore, deep learning models are continuously being optimized and innovated, and datasets are being continuously improved. Autocoders [14], restricted Boltzmann machines (RBM) [12], artificial neural networks (ANN) [15,16], recurrent neural networks (RNN) [17,18], and convolutional neural networks (CNN) [19,20], etc., have been widely used in intelligent detection of the health of machinery. For instance, An et al. [21] developed a self-learning transferable neural network (STNN) based on the learning strategy of humans, which was used for the intelligent diagnosis of machinery fault with unlabeled and imbalanced data. In another study, Chen et al. [22] introduced an enhanced local fusion generative adversarial network to handle the problem of limited training samples and effectively merge representative and diverse information. Furthermore, Chen et al. [23] put forward a dual adversarial guided unsupervised multidomain adaptation network (DAG-MDAN) that utilized an edge adversarial module (EA-Module) to compute the adversarial loss between the source and target domains in each subnetwork. Additionally, an inner adversarial module (IA-Module) was constructed to extract the common features from multiple source domains, enhancing domain confusion through dual adversarial training. Xia et al. [24] proposed an NSDAE-based model with improved activation and cost functions. Song et al. [25] proposed a strategy of widening the convolution kernel to obtain a larger receptive field. On the basis of this, they proposed the convolutional neural network with wide convolution kernels (WKCNN) model, which uses the convolution kernel of the first two convolution layers to quickly extract the features to improve efficiency, and uses smaller convolution cores to achieve multilayer nonlinear mapping to deepen the network and improve the detection accuracy. Osman et al. [26] proposed a new normalized Hilbert–Huang transform (NHHT) technique for the detection of bearing faults. However, in practical applications under such time-varying conditions, time-varying velocity and acceleration can lead to feature smearing, making it difficult to obtain training datasets. Therefore, it is difficult to use data-driven models for data. To train a fault diagnosis model at different speeds, it is necessary to collect training samples at different speeds and accelerations, which is a huge workload.

For deep learning models, the demand for a large, annotated dataset has always been an issue. Especially for bearing fault data [10], the data have the characteristics of a high operating speed, multiple fault types, and a large volume. Moreover, due to the time-varying power and load of mechanical equipment, rotating machinery often operates under time-varying conditions. It is very difficult to collect sufficient samples under time-varying speed conditions, as the superposition of the two-dimensional working conditions of speed and acceleration will make the training samples contain a vast amount of information. This will lead to a sharp increase in the difficulty of intelligent fault diagnosis. An et al. [27] utilized the commonly used infinitesimal method in mathematics and engineering to treat the changing speed as a concatenation of a large number of constant speed segments, ignoring the influence of acceleration. They successfully reduced the dimensionality of the data and proposed a new intelligent fault diagnosis framework. Firstly, the samples are segmented, and the dimensions of each segment are extended through the input network to ensure sufficient information storage space. Secondly, the classification information is stored in the LSTM [28] for transmission. During this process, due to the function of the gates, the information on the working condition is ignored. Finally, the classification of the health status is provided through the output network.

However, the research above may have two drawbacks. Firstly, the large number of LSTM gates requires more parameters. This will result in a longer operation time and overfitting. Secondly, due to the contingency of the dropout [29] method of the training model, the training process will not correspond to the testing process, which is not conducive to improving the accuracy of the diagnostic model. In response to these issues, this article proposes several measures of improvement. (1) In response to the first issue, this study used GRU [30] instead of LSTM to address it. GRU has the same functionality as LSTM, but with a smaller number of gates. While improving the computational speed, it is beneficial for solving the problem of overfitting. (2) In response to the second issue, this study removed the dropout operation and added a residual neural network [31] in front of the GRU network. This helped the model extract features from the data and improve the accuracy of prediction.

The rest of the article is structured as follows. Section 2 provides a detailed introduction to the theoretical foundations related to this study. Section 3 elaborates on the proposed method’s model framework and analyzes the training strategies. Section 4 discusses the selection of the proposed model parameters through experiments. The proposed method was compared with other methods to verify its superiority. Finally, the conclusions are drawn in Section 5.

## 2. Theoretical Background

### 2.1. Convolutional Neural Networks

A convolutional neural network (CNN) is a type of feedforward artificial neural network that can automatically extract the features from data and use them for classification or prediction. The core idea of CNN is to extract the features layer by layer, using multiple basic layer structures for feature extraction and classification, thus achieving efficient and high-precision classification tasks. The basic structure of CNN consists of three parts: the convolution layer, the pooling layer, and the fully connected layer.

(1) The convolutional layer is a core component of CNN and a fundamental operation for feature extraction. It extracts the features from the data by performing convolution operations on the filter (also known as the convolutional kernel or weight) and the original data. Convolutional layers typically include multiple convolutional kernels to extract different features.
(2) The pooling layer is designed to reduce the size and number of features, thereby reducing the computational complexity and further improving the efficiency of feature extraction. The pooling layer is usually used to reduce the dimensions of sparse features and reduce overfitting. There are two commonly used pooling operations: max pooling and average pooling. The maximum pooling operation refers to selecting the maximum value in each region as the output, while average pooling outputs the average value within the region.
(3) The fully connected layer maps the features into feature vectors, which are input into the classifier to achieve the classification of output labels. Usually, a fully connected layer contains multiple hidden units, which can flexibly control the complexity and fitting ability of the model.

### 2.2. Residual Networks

The residual block is the core of the residual network, which contains multiple convolutional layers. The convolutional layer is responsible for extracting the features of the input of residual blocks. Unlike traditional CNN models, the ResNet model incorporates a shortcut connection [31] structure. Through the shortcut connection, the original input information skips over multiple convolution layers and is directly transmitted to the subsequent layers. The output of the convolution layer is taken as the input and activated with the activation function to obtain the output result of this residual learning module. Essentially, it is the difference between the output result and the input, known as the residual. The module contains two types of mapping. The first type is identity mapping; the second is residual mapping *F*(*x*), which ultimately outputs the objective function *H*(*x*) = *F*(*x*) + *x*.

In the ResNet model, the input data EW obtained through several convolutional layers (assuming two layers in this case)

(1) $$F=W_{2}R(W_{1}x)$$

where *W_i_* represents the weight and *R*(*·*) is the ReLU activation function. It is connected to a shortcut, and then, through the nonlinear function ReLU, the final output objective function is:

(2) $$H=F(x,\{W_{i}\})+x$$

When the input’s dimensions are inconsistent with the output’s dimensions, the model performs a linear transformation *W_s_* on *x* at the shortcut, and the final output objective function is:

(3) $$H=F(x,\{W_{i}\})+W_{S}x$$

The residual neural network only learns the residual between the input and output, simplifying the learning objective. Thus, the powerful learning ability of multilevel CNNs can be better utilized, deepening the number of network layers while avoiding the degradation of the network’s performance.

### 2.3. Infinitesimal Method

The infinitesimal method is a common method used to solve mathematical, physical, and engineering problems. Calculus in mathematics and finite element methods in engineering are all based on this idea. Taking calculus as an example, when solving the area enclosed by the function’s curve and the coordinates’ axis, a graph is divided into infinitely small rectangles, and the areas of these rectangles are then summed. In this process, it can be seen that there are two basic steps in the infinitesimal method: splitting and aggregation.

**Figure 1:**
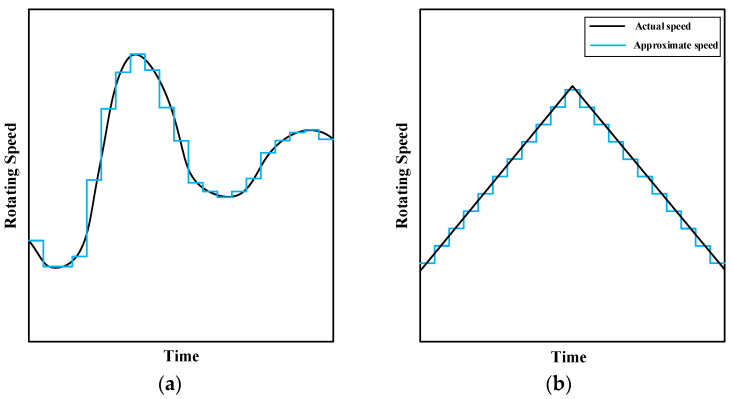
Approximate rotational speed based on the infinitesimal method: (**a**) curve with time-varying speed, (**b**) curve with a constant change in speed.

In the diagnosis of bearing faults under time-varying working conditions, the application of the infinitesimal method can be explained as shown in Figure 1. The speed variation curve of the bearings under time-varying operating conditions can be represented by Figure 1a. At any moment, the changes in velocity and acceleration are random, which brings huge difficulties to the training of the model. The speed variation curve of a uniformly rotating bearing can be represented by Figure 1b. It can be seen that the diversity of the samples is very poor. The sample only has two acceleration conditions. With the infinitesimal method, the time-varying speed change curve can be regarded as a combination of many uniform speed change curves. Therefore, uniform speed samples can be appropriately segmented to train the model. The trained model can diagnose samples with any changes in speed or acceleration within this speed range because the sample with uniform speed contains information about all segments.

According to the concept of the infinitesimal method, the sample size after segmentation is required to be very small, which means that it contains little information. This is not conducive to classifying information, so it is necessary to design a model that has both the ability to split the sample into segments and the ability to aggregate the segments’ information. This can be achieved with a recurrent neural network. A recurrent neural network can continuously extract the microsegments’ sequences, remember the classification information, and finally output the results. This is exactly consistent with the operational process of the proposed infinitesimal method.

### 2.4. RNN

**Figure 2:**
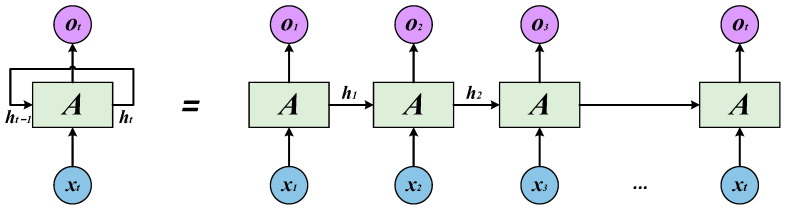
Structure of the RNN.

Unlike traditional neural networks such as ANN and CNN, the recurrent neural network (RNN) is a type of neural network with short-term memory capability. It can allow the internal state information to be transmitted across the network, thereby establishing dependencies between the data. Its structure is shown in Figure 2.

In Figure 2, each neural network module reads the *x_t_* and the output *h_t_*_−1_ of the previous hidden layer, outputting a value of *o_t_*. In a recurrent neural network, the current output of a sequence is also related to the previous output. This is because the nodes between the hidden layers are connected, and the input of hidden layers includes not only the output of the input layer but also the output of the previous hidden layer. Therefore, the output of an RNN at a certain moment is influenced by both the current information and the historical state information, which can be used to fully explore the information contained in time series signals and make predictions related to time series data. However, in practical applications, RNNs may encounter serious long-term dependency problems and may experience “gradient vanishing” or “gradient explosion” when training models using gradient descent methods.

### 2.5. LSTM and GRU

**Figure 3:**
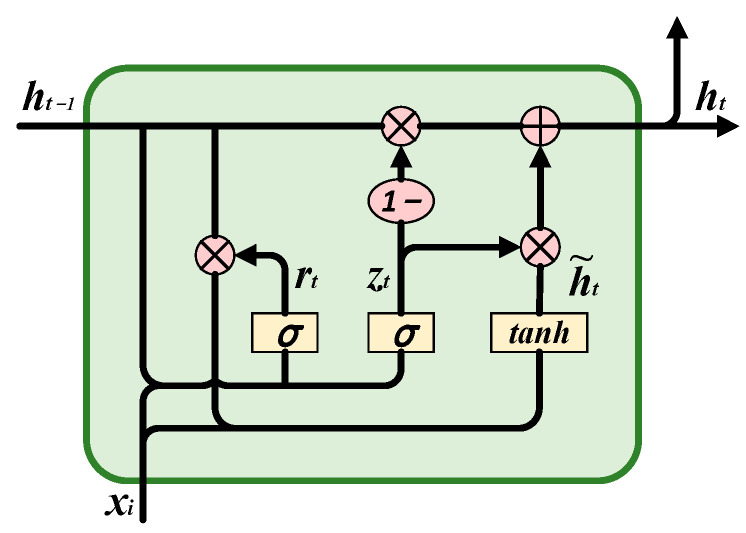
Structure of a GRU cell.

The LSTM and GRU are variants of the RNN. Compared with the RNN, there have been changes in the structure and operations of cells in the GRU and LSTM. The GRU and LSTM have a specifically designed gate structure to replace the hidden units in the RNN, thereby avoiding long-term dependency issues. Note that that long-term information is the default behavior of the GRU and LSTM in practice, rather than an ability to be acquired at a high cost. The difference between these two is that the GRU replaces the input gate, the forgetting gate, and the output gate of the LSTM with the update gate and the reset gate, ensuring accurate predictions while reducing the training parameters. This is not only beneficial for preventing overfitting, but also can achieve a faster convergence speed. The structure of GRU is as shown in Figure 3.

(1) Reset gate: At time step *t*, the GRU network receives the input *x_t_* and the previous hidden state *h_t_*_−1_, and then calculates the reset gate *r_t_*. The symbol *σ* indicates the sigmoid activation function. Its function is to control how much information from the past needs to be retained, and the calculation formula for resetting the gate is

(4) $$r_{t}=\sigma (W_{xr}x_{t}+W_{hr}h_{t-1}+b_{r})$$

where the symbol *W_i_* represents the weight matrix and *b_i_* represents the corresponding bias vector.

(2) Update gate: Its function is to control how much information from the past and current inputs needs to be combined. The calculation formula for updating the gate is:

(5) $$z_{t}=\sigma (W_{xz}x_{t}+W_{hz}h_{t-1}+b_{z})$$

(3) Candidate hidden state: After resetting the information state of the previous time state information *h_t_*_−1_ by resetting the gate *r_t_*, this is activated with the current time information *x_t_* through the function tanh. The specific calculation expression is as follows, where • represents the dot product:

(6) $${\tilde{h}}_{t}=\tanh[W_{x\tilde{h}}x_{t}+W_{h\tilde{h}}(r_{t}•h_{t-1})+b_{\tilde{h}}]$$

(4) Hidden layer state: This controls the output jointly by updating the gate and the candidate hidden layer states. The calculation expression is:

(7) $$h_{t}=(1-z_{t})\cdot h_{t-1}+z_{t}•{\tilde{h}}_{t}$$

It can be seen from the formula above that all gates are vectors activated by the sigmoid activation function. Therefore, all elements of the gate are within the range of (0, 1). At the same time, the operation of the gates is in the form of dot products. This indicates that the gate control unit controls the pass rate of the information and has the characteristic of a “gate”. Based on this special structural design, GRU networks can effectively control the flow of information and retain important information, thereby achieving better performance in processing temporal data.

## 3. Proposed Method

**Figure 4:**
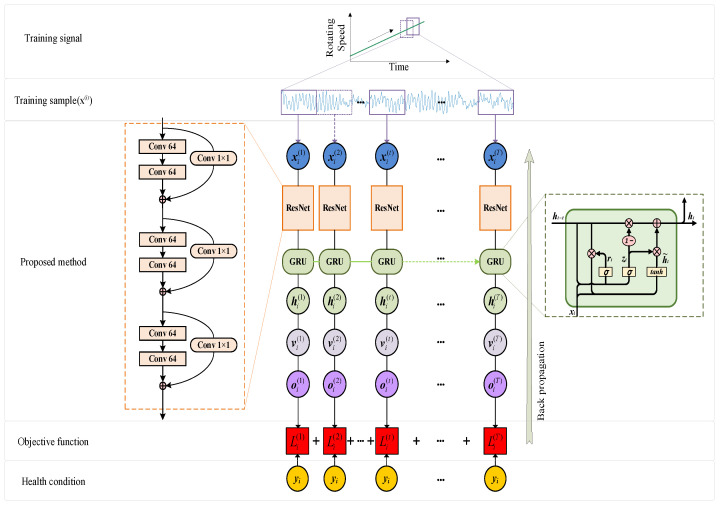
Fault diagnosis methods under time-varying operating conditions.

Figure 4 shows the framework of the method proposed in this article. It is a fault diagnosis method based on the ResNet and GRU as the core. This section will elaborate on its specific content.

### 3.1. Training Dataset

Based on the infinitesimal method, the proposed model can ignore the differences in acceleration. Therefore, the training data only require diverse speed information. The collected signal is long, and when preprocessing the data, repeated sampling is used for simple data enhancement, with an overlap rate of 50%. The final training set ${\left\{x_{i},\;y_{i}\right\}}_{M\;i=1}$ for the model is obtained, where *M* is the number of samples and *x_i_*∈R*^N^*^×1^. *y_i_* = 1, 2, …, *C* is the label for *x_i_*. Then *N* represents the dimensions of the sample and *N_in_* represents the input dimensions of the model. Each sample *x_i_* is divided into a fragment set ${\left\{x_{(t)i}\right\}}_{T\;t=1}$, where *T* = *N*/*N_in_*.

### 3.2. Information Expansion and Feature Extraction

The model first utilizes residual neural networks to extract the features from the signal. Three residual blocks are used here. The working process of each residual block is as follows. Firstly, the input data are extracted through a convolutional layer for feature extraction. The convolution layer has 64 output channels, the size of the convolution core is 3, ReLU is used as the activation function, and the padding is set to “same” to keep the input and output lengths the same. This step aims to capture the local features of the input data. Next, it adjusts the number of output channels to 64 through a 1 × 1 convolutional layer for adding to subsequent features. Then a convolutional layer with the same parameters as the first convolutional layer is used again, which further enhances the feature expression ability. Afterwards, the addition layer adds the output of the previous convolutional layer and the previously saved residual (i.e., the output of the 1 × 1 convolutional layer) to form a residual connection. The purpose of doing so is to learn the residual part in order to better preserve the original features. According to the concept of the infinitesimal method mentioned earlier, the proposed model should be able to handle small fragments. However, the small dimensionality of the model input also means that there is very little stored information. Therefore, the residual network also plays a role in expanding the dimensions of the original small fragments. This ensures that the hidden layer in the GRU can store enough information.

### 3.3. GRU Layer

The GRU is the core of this method. The update gate first performs a sigmoid nonlinear mapping of the input *x_t_* and the previous hidden layer output *h_t_*_−1_ as the information to be retained. The larger the *z_t_* value, the more state information is retained from the previous moment to the current moment. On the contrary, 1 − *z_t_* is the information to be discarded. The model resets the gate to perform sigmoid nonlinear mapping of *x_t_* and *h_t_*_−1_. The obtained result is multiplied by *h_t_*_−1_ and subjected to a tanh change, determining the degree to which the previous information is forgotten. Through this gating mechanism, GRU networks can effectively control the flow of information and retain important information.

### 3.4. Output Network

The output network consists of two layers of the artificial neural network, with the weight matrices *V*_1_ and *V*_2_, respectively. The final output feature *o*^(*T*)^ is a vector containing *C* softmax classification units, and *C* is the number of health conditions. The posterior probability of the sample belonging to each category *c* will be given, and the formula is as follows:

(8) $$P(y=c\left|x\right.)=\frac{e^{o_{\left[c\right]}^{\left(T\right)}}}{\sum_{j=1}^{C}e^{o_{\left[j\right]}^{\left(T\right)}}}$$

In the equation, [*c*] and [*j*] represent the elements of the output vector, and the final output vector *o*^(*T*)^ is obtained by the following formulas

(9) $$v^{\left(T\right)}=\mathrm{ReLU}(V_{1}h^{\left(T\right)}+b_{v1})$$

(10) $$o^{\left(T\right)}=V_{2}v^{\left(T\right)}+b_{v2}$$

where *b_v_*_1_ and *b_v_*_2_ are the bias vectors of the output network, and the dimensions of the feature layer *v*^(*T*)^ equal 50.

### 3.5. Loss Function

The calculation of the loss function refers to the method in Reference [27]. After all the fragments ${x_{\left(t\right)}}_{i}$ are sequentially input into the model, the corresponding output fragment set ${\left\{o_{(t)i}\right\}}_{T\;t=1}$ will be obtained. In traditional recurrent neural networks, only the last output segment $o_{(T)i}$ participates in training. However, this method proposes a loss function based on the overall sequence, which involves the entire output fragment set in training to ensure strong supervised training at each time point. Therefore, each output segment $o_{(t)i}$ is combined with its label *y_i_* and trained using the softmax loss function.

(11) $$L_{i}^{\left(t\right)}=-\sum_{c=1}^{C}1\left\{y_{i}=c\right\}\log\frac{\exp\left(o_{i,\left[c\right]}^{\left(t\right)}\right)}{\sum_{j=1}^{C}\exp\left(o_{i,\left[j\right]}^{\left(t\right)}\right)}$$

where 1 {•} represents the indicator function, that is, when the equation is established, it returns 1; otherwise it is 0. The final loss function is the union of each loss function fragment:

(12) $$L=\frac{1}{M}\sum_{i=1}^{M}\sum_{t=1}^{T}L_{i}^{\left(t\right)}$$

### 3.6. Optimization

All weight matrices *W*, *V*, and their corresponding bias vectors *b* are trained using backpropagation algorithms. The adaptive motion estimation algorithm (Adam) is used to minimize *L*. The trained model can determine the health status of each time step *t* under any changing speed conditions based on the output fragment $o_{(t)i}$.

## 4. Case Study on the Diagnosis of Rolling Bearing Faults under Time-Varying Rotational Speeds

### 4.1. Data Description

**Figure 5:**
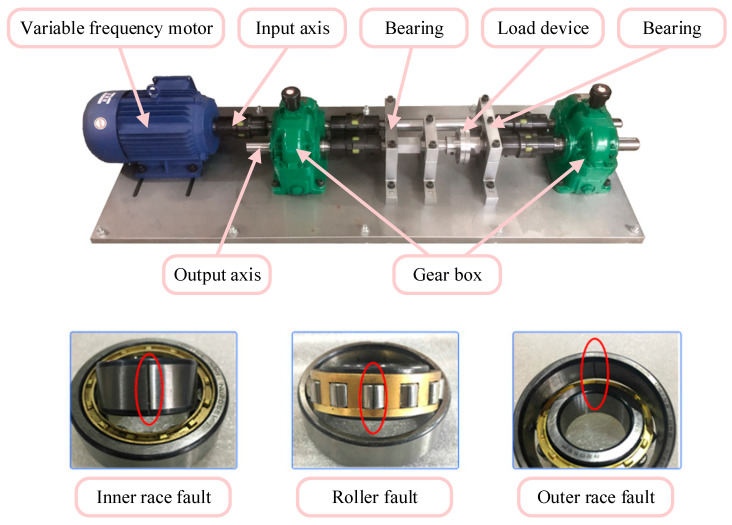
Time-varying speed bearing fault experimental bench and faulty bearing.

The data used in this section come from the experimental platform shown in Figure 5. The experimental platform consisted of a variable frequency motor, an input shaft, an output shaft, a gearbox, bearings, and the load device. The acceleration sensor was placed on the bearing seat. The motor’s speed range was 0–1500 rpm, and the sampling frequency was 12.8 kHz. The data included three types of working conditions:

(1) Uniform acceleration: The motor accelerated from stationary to a maximum speed of 1500 rpm. The acceleration remained constant throughout the entire process. The model used samples under this operating condition as the training samples. However, in practical applications, the proposed model does not require the rate of change in speed to remain constant but instead takes a certain speed range.
(2) Random speed changes: The speed and rate of change of the equipment during operation are often irregular. Therefore, this sample was mainly used to simulate the actual situation of a time-varying rotational speed and served as a test sample in this experiment.
(3) Constant speed conditions: The rate of the change in speed of the constant-speed sample was 0, so it was significantly different from the training sample. The experimental platform collected vibration signals at a speed of 1000 rpm to test the effectiveness of the generated data.

Each dataset contained five health conditions. The bearings were preset with three single faults, namely an inner race fault (IF), a rolling element fault (RF), and an outer race fault (OF), as well as a composite fault of outer race and roller element faults (ORF). In addition, there were bearings in a normal condition (NC). There were 2000 samples for each health condition. The sample size was 1200, with a total of 10,000 samples.

### 4.2. Effects of Hyperparameters

In the model proposed in this article, there are three key parameters, namely the input dimension, the number of residual network layers, and the dimensions of the hidden layer of the GRU. The following experiments investigated the impact of different parameters on the model’s performance. All experiments were repeated 10 times to avoid the contingency of model training.

**Figure 6:**
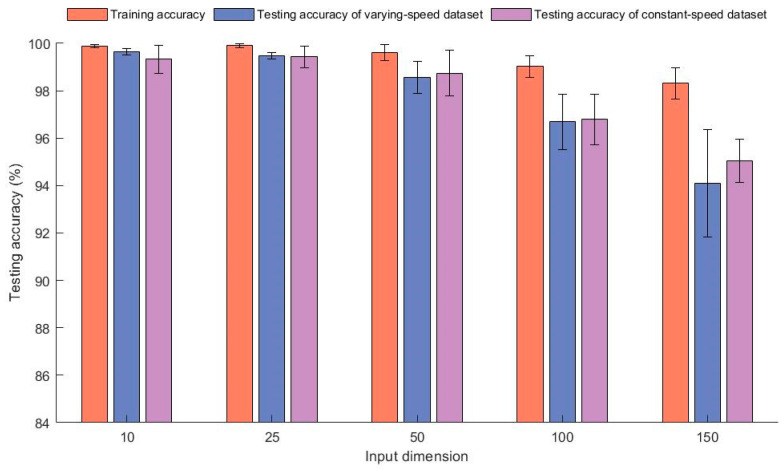
The model’s diagnostic accuracy and standard deviations under different input dimensions.

The average diagnostic accuracy of the model under different input dimensions is shown in Figure 6. In the experiment, the residual neural network had 12 layers. When the input dimension was less than 100, the dimensions of the hidden layer of the GRU equaled 100. When the input dimension was greater than 100, the dimensions of the hidden layer of the GRU network were set to 200. From the graph, it can be seen that when the dimension was less than 50, the training accuracy reached over 99.5% and the testing accuracy reached over 98.5%. From this, it can be seen that this model could diagnose fault samples with large fluctuations in speed. As shown in the figure, when the input dimension was 10 and 25, the testing accuracy was very high, but when the dimension was greater than 25, the training accuracy and testing accuracy showed a downward trend as the dimension increased. This further validated the infinitesimal method on which the model is based. When the fragments’ size was large, the differences in the acceleration information contained in it could not be ignored, which led to a decrease in the accuracy of the model’s diagnosis. In addition, the smaller the input dimension, the longer the time required for model training. Thus, when the accuracy is high, we should choose parameters with a shorter training time. Therefore, we selected 25 as the input dimension.

**Figure 7:**
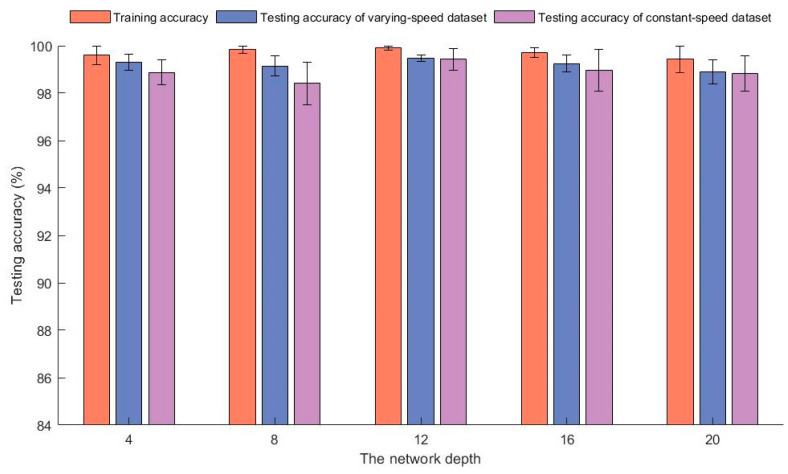
The model’s diagnostic accuracy and standard deviations with different residual network layers.

The average test accuracy with different residual network layers is shown in Figure 7. In the experiment, the input dimension was 25 and the dimension of the GRU layer was 100. From the graph, it can be seen that the model’s training accuracy with different residual network layers could reach over 99.4%. When the number of residual network layers was 12, the accuracy for variable speed testing and uniform speed testing was higher. When the number of residual layers was less than 12, the accuracy of testing significantly decreased. This is because the learning ability of the residual network was limited when the number of layers was low. The fitting ability of the model was insufficient, resulting in underfitting. When the number of residual layers was greater than 12, the testing accuracy also decreased. As the number of residual layers increased, the number of parameters to be trained increased, and the generalization ability of the model began to decrease. According to the analysis above, 12 was selected as the number of residual network layers.

**Figure 8:**
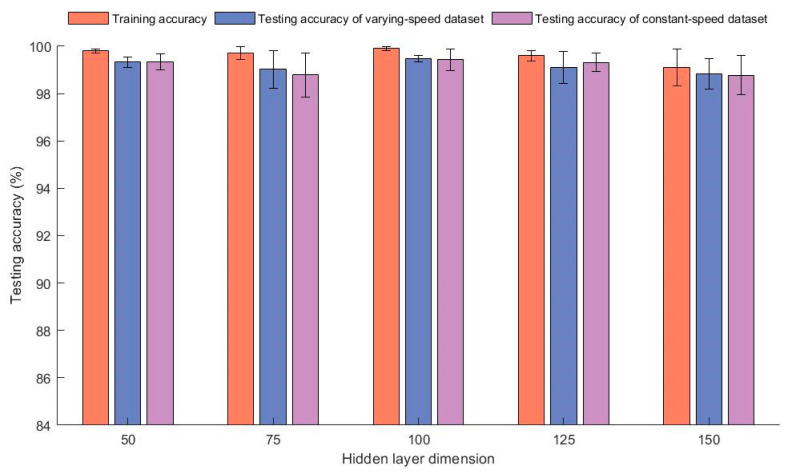
Diagnostic accuracy and standard deviation of models with different dimensions of the GRU’s hidden layers.

The average diagnostic accuracy of the model with different dimensions of the GRU layer is shown in the Figure 8. In the experiment, the input dimension was 50 and the number of residual network layers was 12. The figure shows that the testing accuracy of all models was above 98.7% and there was not much difference in the results in various situations. From this, it can be seen that the dimension of the GRU layer has a small impact on the prediction accuracy of the model, so a dimension of 100, which had the highest testing accuracy, was selected.

At this point, the model’s structure and key parameters of each layer are discussed. Table 1 presents a more intuitive visualization of this information.

### 4.3. Comparison with Related Methods

To demonstrate the superiority of the proposed method, other commonly used methods were compared with the proposed method. In each experiment, the model used the same training and testing data. When applying the ANN method, the spectrum of the sample was used as its input, and a five-layer neural network was used. The cost function adopted the standard softmax function. The activation function of each layer was ReLU, and the learning rate was 0.001. For CNN, the method described in Reference [4] was used. When applying DBN, the method described in Reference [32] was used. For LSTM networks, the method described in Reference [27] was used. Using the spectrum as the input, a four-layer network structure was adopted. The first layer used an artificial neural network to expand the dimensionality of the data. The second layer was an LSTM network with a hidden layer dimension of 100. The third and fourth layers were two-layer artificial neural networks with structures of 50.5 each.

**Figure 9:**
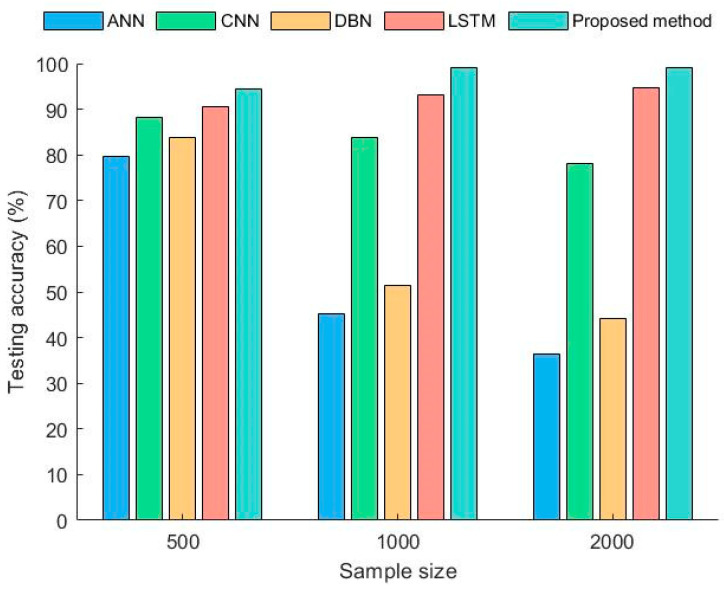
Diagnostic accuracy of different methods under time-varying rotational speeds.

**Figure 10:**
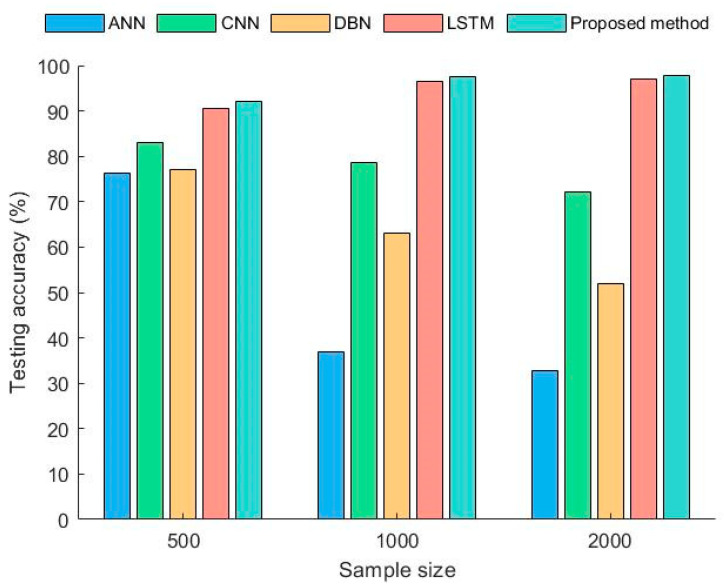
Diagnostic accuracy of different methods under uniform rotational speeds.

To study the impact of different sample sizes on the model, three datasets were designed with sample sizes of 500, 1000, and 2000. The training dataset for all methods was a uniform acceleration dataset, while the testing datasets were a time-varying speed dataset and a uniform speed dataset. Each experiment was conducted 10 times, and the average accuracy obtained is shown in Figure 9 and Figure 10.

From the Figure 9, it can be seen that each deep learning method had high testing accuracy when the sample size was 500. However, as the sample size increased, the testing accuracy of ANN and DBN decreased significantly. The decrease found for CNN was small, but when the sample size reached 2000, the testing accuracy was less than 80%. LSTM and the proposed method performed well, with a diagnostic accuracy rate of over 90% in all cases. The accuracy of the proposed method was significantly higher than that of LSTM.

Due to the significant differences between the uniform speed samples and the training samples, the accuracy of each method decreased to varying degrees. The diagnostic accuracy of ANN, CNN, and DBN still decreased with an increase in the sample size. This indicates that the traditional deep learning methods could not ignore the differences in acceleration between the training and testing samples. This led to a larger sample size and lower diagnostic accuracy. The LSTM model constructed in line with the infinitesimal method, to some extent, solved this problem, but there was still a certain gap in accuracy compared with the proposed method. Therefore, the proposed method not only breaks the limitations of traditional deep learning methods but also has better diagnostic capabilities compared with diagnostic models that rely solely on LSTM as the core.

To further demonstrate the superiority of the proposed method, Table 2 lists the highest accuracy in diagnosing faults under time-varying conditions via different methods in the experiments above. An advanced fault diagnosis method, AIDA, has been added here. AIDA [33] is a data augmentation method which can increase the ability to the extension of CNN. From Table 2, it can be seen that the proposed method had the highest accuracy value. It can be seen that the model had good adaptability and processing ability for diagnosing bearing faults under time-varying working conditions.

### 4.4. Network Visualizations

In order to gain a clearer understanding of the role played by each layer in the model, t-SNE was used to study the feature characteristics of each layer at different time steps. T-distributed stochastic neighbor embedding (t-SNE) is a nonlinear dimensionality reduction technology used to map high-dimensional data to low-dimensional spaces for visualization and clustering analyses. T-SNE can map high-dimensional data to a low-dimensional space. During this process, t-SNE preserves the local structure between the data, meaning that similar data in the original high-dimensional space should also be similar in the low-dimensional space. By observing the results of visualization, we can determine whether the model could correctly distinguish samples from different categories, or whether there were instances of category overlap or confusion. For the convenience of research and analysis, samples with a uniform rotational speed were used. Because the information on the operating conditions of the samples with a uniform rotational speed is very stable, it can fully reflect the differences in the operating conditions. However, there is inevitably an overlap in the speed information of samples with a time-varying rotational speed, which is not conducive to research.

**Figure 11:**
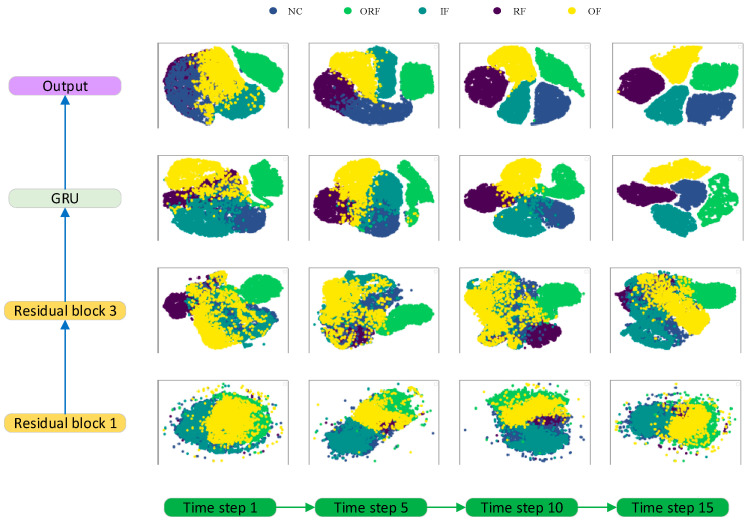
Visualization of the principal components of the features at each layer.

Figure 11 uses t-SNE to reduce the features to two dimensions. From the figure, it can be seen that after passing them through a residual block, it was still difficult to distinguish the fault features. After three residual blocks, the distinguishability of the faults’ features significantly improved. This indicates that it is necessary to increase the depth of the residual blocks. However, it can be seen that the ability of the residual networks was limited. After passing through the GRU layer, the discrimination of the faults’ features was qualitatively improved. As the time step increased, feature differentiation gradually became apparent. This indicates that this layer of the neural network accumulated information. When reaching the output layer, obvious, discriminative, and stable features were extracted.

## 5. Conclusions

This article proposes a method of diagnosing bearing faults under time-varying operating conditions. This method ignores the impact of acceleration on the sample through the infinitesimal method, reducing the dimensionality of the data. The model’s framework includes three main parts. Firstly, feature extraction is performed on the data through ResNet. Secondly, the information is input into the GRU, and the gate structure of the GRU filters and accumulates the information. Finally, the classification results are output through an output network composed of two layers of artificial neural networks. Through experiments, the influence of different parameters on the model was discussed. Furthermore, the optimal parameter selection for the model was determined. Comparative experiments showed that this method had better diagnostic capabilities than the existing methods and it was very suitable for diagnosing bearing faults under time-varying working conditions. Finally, the study analyzed the functions of each layer of the model using the t-SNE method. This study improved the application of the infinite element method in diagnosing bearing faults under time-varying conditions. An effective solution is provided for the problem of difficulty in diagnosing time-varying operating conditions using data-driven methods. When the diversity of the training data’s conditions is further reduced, such as in the case of a single speed or several speeds, the performance of the proposed model decreases. Research on this issue will be the focus of future work.

## Figures and Tables

**Table 1:** Parameter set of each layer.

Network Layer	Number and Size of Convolution Kernels	Activation Function	Output Size
Input	/	/	48 × 25 × 1
1D convolution 1	3 × 1 × 32	ReLU	48 × 25 × 32
1D convolution 2	1 × 32 × 64	/	48 × 25 × 64
1D convolution 3	3 × 64 × 64	ReLU	48 × 25 × 64
Addition layer (2 + 3)	/	/	48 × 25 × 64
1D convolution 4	1 × 64 × 64	/	48 × 25 × 64
1D convolution 5	3 × 64 × 64	ReLU	48 × 25 × 64
1D convolution 6	3 × 64 × 64	ReLU	48 × 25 × 64
Addition layer (4 + 6)	/	/	48 × 25 × 64
1D convolution 7	1 × 64 × 32	/	48 × 25 × 32
1D convolution 8	3 × 32 × 32	ReLU	48 × 25 × 32
1D convolution 9	3 × 32 × 32	ReLU	48 × 25 × 32
Addition layer (7 + 9)	/	/	48 × 25 × 32
1D convolution 10	3 × 32 × 1	ReLU	48 × 25 × 1
GRU	/	/	48 × 25 × 1
Full connection	50	ReLU	48 × 50 × 1
Full connection	5	Softmax	48 × 5 × 1

**Table 2:** Highest accuracy of different methods.

Method	Highest Accuracy
ANN	80.9%
CNN	89.2%
DBN	85.1%
LSTM	95.3%
AIDA	97.8%
Proposed method	99.5%

## Data Availability

Not applicable.
